# Critical Care Physicians’ Perspectives on Nudging in Communication

**DOI:** 10.1001/jamanetworkopen.2025.31199

**Published:** 2025-09-10

**Authors:** Derek R. Soled, Christy L. Cummings, Laura M. Berbert, David N. Williams, William B. Feldman, Robert D. Truog, Emily B. Rubin

**Affiliations:** 1 Department of Medicine, Brigham and Women’s Hospital, Boston, Massachusetts; 2 Department of Pediatrics, Boston Children’s Hospital, Boston, Massachusetts; 3 Harvard Medical School, Boston, Massachusetts; 4 Division of Newborn Medicine, Boston Children’s Hospital, Boston, Massachusetts; 5 Center for Bioethics, Harvard Medical School, Boston, Massachusetts; 6 Biostatistics and Research Design Center, Boston Children’s Hospital, Boston, Massachusetts; 7 Division of Pulmonary and Critical Care Medicine, Brigham and Women’s Hospital, Boston, Massachusetts; 8 Program on Regulation, Therapeutics, And Law, Division of Pharmacoepidemiology and Pharmacoeconomics, Brigham and Women’s Hospital, Boston, Massachusetts; 9 Division of Critical Care Medicine, Boston Children’s Hospital, Boston, Massachusetts; 10 Division of Pulmonary and Critical Care Medicine, Massachusetts General Hospital, Boston

## Abstract

**Importance:**

Research in behavioral economics has demonstrated that people have irrational biases, which make them susceptible to decisional shortcuts, or heuristics. The extent to which physicians consciously might use nudges to exploit these heuristics and thereby influence their patients’ decision-making is unclear. In addition, ethical questions about the conscious use of nudges in medicine persist, yet little is known about how physicians experience and perceive their use.

**Objective:**

To explore critical care physicians’ perspectives on nudging to guide patients in clinical decision-making.

**Design, Setting, and Participants:**

This qualitative study involved semistructured interviews of critical care physicians at 3 tertiary care hospitals in Boston conducted between June and September 2024. Adult, pediatric, and neonatal critical care attending physicians were randomly selected to participate in the study. Transcripts were analyzed using qualitative thematic analysis from October 2024 to February 2025.

**Main Outcomes and Measures:**

Themes and subthemes that reflected physicians’ experiences and views.

**Results:**

A total of 100 physicians were invited, and 54 physicians (29 [53.7%] male) were interviewed; 35 (64.8%) were adult intensivists, 13 (24.1%) were pediatric intensivists, and 6 (11.1%) were neonatal intensivists. Four main themes were identified: (1) nudging was generally seen as a positive and necessary part of medical communication; (2) physicians had ethical concerns about nudges, especially relating to autonomy; (3) nudging may be more effective and appropriate in certain situations but counter-productive in others; and (4) physicians’ experiences and perspectives varied by practice setting, sex, and experience. Adult intensivists engaged in more direct recommendations and transparent nudges, while pediatric and neonatal intensivists supported more subtle nudging to foster shared decision-making. Female physicians tended to use nudges to emphasize patient and family emotional well-being, while male physicians tended to highlighted efficiency and outcomes as the primary reasons to nudge; junior physicians were more hesitant to use nudges compared with their more senior counterparts.

**Conclusions and Relevance:**

In this qualitative study, physicians acknowledged that it was important to use nudges when communicating with patients, while noting ethical uncertainty. These findings have implications for how nudging may be used as an effective and appropriate communication technique depending on context.

## Key Points

**Question:**

What are the experiences and perceptions of critical care physicians in using nudges to frame medical choices and thereby guide patient decision-making without limiting alternative options?

**Findings:**

In this qualitative study of 54 critical care physicians from 3 hospitals in Boston, clinicians viewed nudging as a natural part of medical communication that could improve patient outcomes. However, most physicians raised ethical concerns about nudging, which varied by practice setting, sex, and experience.

**Meaning:**

In this study, physicians reported that although use of nudges may be a necessary, effective, and sometimes appropriate mode of communication in intensive care units, ethical questions remain.

## Teaser

This qualitative study explores critical care physicians’ perspectives on nudging to guide patients in clinical decision-making.

## Introduction

Research in behavioral economics has expanded our understanding of decision-making and demonstrated that people have irrational biases, which may make them susceptible to decisional shortcuts, or heuristics—cognitive processes that help the brain make decisions quickly and efficiently with minimal mental effort. In the decision-making context, a *nudge* is defined as “any aspect of the choice architecture [the intentional arrangement and presentation of options to subtly guide people towards certain choices] that alters people’s behavior in a predictable way without forbidding any options or significantly changing their economic incentives.”^1^ While nudges preserve a person’s choice set without restricting options, they make it more likely that a person will choose some particular option by triggering decision-making heuristics and biases.^2,3,4^

In medicine, a nudge might be when a physician frames information with a positive or negative slant (eg, presenting treatment benefits in terms of relative risks instead of absolute terms affects rates of breast cancer treatment^5^), highlights what other patients in similar situations might do (eg, emphasizing the number of patients who have received the human papillomavirus vaccine is associated with the likelihood of a patient receiving it^6^), offers options as a default (eg, when end-of-life care is defaulted to no aggressive treatments, patients are more likely to decline cardiopulmonary resuscitation^7^), or presents information in a way that highlights potential gains instead of losses (eg, noting that a procedure has a 99% success rate is associated with increased uptake vs saying it has a 1% failure rate^8^). By definition, a direct recommendation would not count as a nudge.

Ethicists have debated the justifiability of using nudges in medicine.^9,10,11,12,13,14,15,16,17,18,19,20,21^ One concern is that nudges may prevent a patient from being fully informed, potentially violating their autonomy. While many studies have demonstrated the efficacy of nudges,^22^ few have assessed physician experiences regarding their use. Moreover, there is minimal research on the use of nudges among adult, pediatric, and neonatal intensive care units (ICUs) or how different clinicians might grapple with their ethical uncertainties. Past studies have focused, for instance, on the use of nudges in particular practice settings, such as family meetings in pediatric ICUs.^23^ In our qualitative study, we seek to better understand physician perspectives on the use and ethics of nudging in clinical practice across adult, pediatric, and neonatal ICUs.

## Methods

### Design

We used a qualitative phenomenological research design^24,25,26,27^ to explore and describe physician experiences and perspectives with nudges. This study was approved by the institutional review boards of Mass General Brigham and Boston Children’s Hospital. All participants provided verbal informed consent. This study followed the Consolidated Criteria for Reporting Qualitative Research (COREQ) reporting guideline for qualitative studies.

### Setting and Participants

This study was conducted at 3 tertiary hospitals in Boston, Massachusetts. Each site’s primary investigator provided the names of all intensivists. A random but purposeful sample of 100 attending physicians (with proportional representation of adult, pediatric, and neonatal intensivists reflecting each site) was selected from those lists and contacted via email. We focused on intensivists (rather than clinicians from other specialties) given the high-stakes nature of many conversations in the ICU and the potential prevalence and utility of nudges in such high-stakes conversations. Ultimately, 54 individuals enrolled and were offered a $50 Amazon gift card for their participation.

### Statistical Analysis

Interviews lasted approximately 45 to 60 minutes and were conducted via secure video conference by a single interviewer (D. R. S.) between June and September 2024 using a semistructured interview guide (eAppendix in Supplement 1). Participants self-reported their demographic information, practice setting, and years of experience. Race and ethnicity were collected as Asian, Black or African American, Hispanic, Middle Eastern, multiethnic, White, and other (ie, individuals who self-reported other race or ethnicity). Interviews were recorded, transcribed, and deidentified. From October 2024 to February 2025, coding and analysis were performed using principles of thematic analysis^28,29^ using Dedoose software version 9.0.107 (Sociocultural Research Consultants). A coding dictionary was created and continuously updated as needed. D. R. S. and L. B. performed line-by-line blinded coding, and discrepancies were discussed and resolved. Data saturation was achieved by interview 40 and confirmed by the analysis team. The team (D. R. S., L. B., and D. N. W.) reached consensus on meaningful themes, subthemes, and conceptual links. We defined a notable difference or trend as when a phenomenon was noted in more than 50% of participants.

## Results

Among 54 physicians (29 [53.7%] men) enrolled and interviewed, 35 (64.8%) were adult intensivists, 13 (24.1%) were pediatric intensivists, and 6 (11.1%) were neonatal intensivists (Table 1). Most participants (45 participants [83.3%]) had no formal training in nudging (defined as attendance in ≥1 class or conference on decision-making science during or after medical school), but all participants reported some informal training on these topics, such as discussions during rounds or throughout their medical training.

**Table 1.** Participant Characteristics

Characteristic	Participants by practice setting, No. (%)		
	Medical ICU	Pediatric ICU	Neonatal ICU
Total	35 (64.8)	13 (24.1)	6 (11.1)
Sex			
Male	21 (60.0)	6 (46.2)	2 (33.3)
Female	14 (40.0)	7 (53.8)	4 (66.7)
Race and ethnicity			
Asian	8 (22.9)	1 (7.7)	0
Black or African American	0	0	0
Hispanic	1 (2.9)	0	0
Middle Eastern	1 (2.9)	0	0
Multiethnic	0	0	0
White	24 (68.6)	12 (92.3)	6 (100)
Other^a^	1	0	0
Age, y			
30-39	19 (54.3)	2 (15.4)	2 (33.3)
40-49	12 (34.3)	5 (38.4)	2 (33.3)
50-59	4 (11.4)	1 (7.7)	0
60-69	0	4 (30.8)	2 (33.3)
70-79	0	1 (7.7)	0
Years in practice			
0-9	12 (34.3)	1 (7.7)	1 (16.7)
10-19	18 (51.4)	3 (23.1)	3 (50)
20-29	4 (11.4)	6 (46.2)	1 (16.7)
30-39	1 (2.9)	2 (15.4)	2 (33.3)
40-49	0	1 (7.7)	0

Abbreviation: ICU, intensive care unit.

^a^ Includes individuals who identified as other race or ethnicity.

Four overarching themes were identified: (1) nudging is a positive and necessary part of medical communication; (2) physicians have ethical concerns about nudges, especially relating to autonomy; (3) nudging may be more effective and appropriate in certain situations but counterproductive in others; and (4) physician characteristics matter. Subthemes emerged under each overarching theme. Representative quotes^30^ for the first 3 themes are presented in Table 2. Trends related to the fourth theme are summarized in Table 3.

**Table 2.** Themes and Subthemes With Representative Quotations

Themes and sub-themes	Quotations (practice setting, identifier)
**Nudging is a natural and necessary part of medical communication**	
Physicians have a responsibility because of their expertise	“I think a lot of people haven’t seen or taken care of someone that sick before, and at that point, our expertise provides another layer of advice, I suspect, and that’s when I feel it’s more ethically appropriate to provide these nudges and our perspectives.” (MICU, 49)
	“I think it is part of our job to convey what we’ve seen and our expertise in some ways to advocate for what we suspect is the right thing.” (PICU, 42)
	“I think our role is to present it in a way that…takes our training and experience and distills it into digestible form in a situation that’s life threatening and often overwhelming.” (MICU, 45)
	“I think it’s my job to guide the families. I’ll give you a classic example that happens all the time…: you offer the family this decision, ‘What would you like to do?’ and they are incapacitated. They can’t make that decision, so you with your infinite expertise and wisdom need to act on their behalf.” (NICU, 42)
Integral to beneficence	“Oh, I think it’s unethical not to do it. I honestly think if I’m like, ‘Hey, whatever you guys decide, we can do whatever,’ I think your job is to help interpret data and communicate to people and to help people navigate complex parts, you know, some of the difficult parts in their lives, and if you just randomly say, ‘We can do whatever,’ like you’re not helping people. You’re just there. You’re a technician, and you’re not really like a healer, and so, yeah, I mean, I think navigating people through—and again, I’m not really trying to nudge them to a specific decision. I’m trying to nudge them, you know, to have the proper motivation, to align their goals with what their goal should be, which is acting on behalf of the patient.” (MICU, 15)
	“I think it gives comfort to the patients in particular what you’ve seen other people do, so I definitely use that one…” (MICU, 7)
	“And I don’t think that patients’ families are, like, frightened of emotion. I think there used to be this, like, notion that like, the physician should be impassive and objective at all times, and I think that’s categorically false. Like, I think that patients want to know that you care about their loved one and that you’re trying what’s best for them even when that’s a hard decision to make and that [through the use of nudging] you’re helping them through that hard decision so that they don’t have to bear it by themselves.” (MICU, 18)
	“I think for families who are really struggling with the decision, like they’re tired, it’s taken a huge toll on their families in ways that they did not imagine when they first made a decision…we give them permission to make the decision that seemed wrong from the first place but is actually the right decision for them…” (PICU, 25)
Builds trust with patients and families	“Shared decision-making takes place more in sort of those gray areas when there is more than 1 reasonable approach, and that’s where it’s really helpful to talk to families and see the values and go from there…nudging can be one of the most effective ways to help facilitate shared decision-making.” (MICU, 48)
	“I feel comfortable that as long as I trust myself, meaning I think I’m showing integrity, I’m showing benevolence, and I know what I’m talking about, you know, I think that my nudge is what they’re expecting. It’s part of the contract.” (MICU, 1)
	“…if the person nudging me has my best interest in their mind, is transparent, and is good at what they do, I’ll take their nudge, and this is part of trust and decision-making…when I’m nudging a patient, and maybe this is why I actually speak out loud—I’m expressing ability, I’m expressing transparency by thinking out loud, and I’m expressing integrity, and it’s up to the patient to make a decision. Is this enough for them to trust me?” (NICU, 28)
	“You’re doing what patients come to physicians for, which is to help them feel good about making the best decision possible for themselves, and that’s why it is so dependent on the context and the situation and your relationship with a patient and their family and your prior conversations with them…to know that you are helping guide them and not actually manipulating them…” (PICU, 19)
Counteracts existing biases	“Part of our role as clinicians is to make a recommendation or give our insight into what we think the most appropriate treatment course is, so if it’s not a direct recommendation, you know, giving nudges to say, ‘Here’s what I might think about in this circumstance. Here are the risks of this scenario. Here’s what the best-case scenario is.’ I think that allows patients to make more informed decisions, because I think that, especially in life-and-death scenarios, most people naturally will choose life and, you know, but what that means realistically down the road, I think many people don’t understand.” (MICU, 9)
	“I think I would use it [nudging] as a way to help them be fully informed and make a set of choices for themselves.” (PICU, 4)
	“…as physicians, it’s our job to synthesize data. I think it’s important for us to do that in a way that keeps track of our implicit biases and try to be impartial in the way that we synthesize data, but giving patients an a la carte menu of resuscitation options and asking them to choose 1 out of context, I think is not the best use of our medical training and communication skills.” (MICU, 54)
	“I would usually say, ‘Well, it seems like you would rather not do something that might have a risk or this big of a risk despite knowing the potential benefit, and so based on that, let me tell you more about this other option and what its risks and benefits might be.’ But I think I try to always be explicit about what I’m inferring about them so that I’m not doing it wrong.” (NICU, 50)
Streamlines the decision-making process	“I tend to give a spectrum of options that are relatively discrete and framed around patient values…in this way I am nudging patients towards only reasonable and likely choices.” (MICU, 14)
	“I constantly reference what the patient would want and their goals so it makes it easier, the whole decision-making process, like it [the nudge] keeps it in the front of their mind that we’re just trying to serve her, not serve us kind of stuff.” (MICU, 15)
	“There are nudges, like our antibiotics time out at 4 days unless we specifically extend them, so, you know, it helps prevent unnecessary use of antibiotics…there’s a lot to keep track of, and so we do have nudges there to kind of remind people, ‘Oh, it’s time to do this or not do that,’ but that’s all built on trying to streamline the care.” (MICU, 41)
	“I think what’s challenging is later on in the course, when it’s very clear that the patient is very ill and is not going to recover. That’s when there aren’t many options, and then I kind of feel it is our obligation to really lay it out that this is what we would advocate as best for the patient…. When it’s a lot more clear, it’s easier to say, ‘This is what we’ve seen many times, and this is likely what’ll happen.’…Us nudging helps the family better understand the most likely trajectory.” (MICU, 7)
**Physicians have ethical concerns about nudges**	
Infringements on patient autonomy and informed consent	“I don’t want to say that nudges are never appropriate, but in general, I think trying to minimize bias in your offering decisions to families that truly should be based on parental preferences should be pursued.” (NICU, 46)
	“My instinct around this is that…nudging is probably not appropriate as a first tack in a conversation. Like that could be a way to set the stage in a certain direction that seems particularly influential and may be overly biased…trying to nudge them seems not optimal…and also seems somewhat unethical, to be honest, in terms of trying to take away some of their autonomy.” (MICU, 26)
	“I’ll tell you my gut reaction to just the concept is that I don’t love it. I think…it’s like a little manipulative…if I know what I think is appropriate, I should be explicit about it.” (MICU, 8)
	“I think it’s less appropriate when someone’s just not making the decision you want them to make, and that’s challenging and sometimes really hard to even know what to say at that point, but I think trying to nudge them seems not optimal in terms of the relationship building tack and also seems somewhat unethical, to be honest, in terms of trying to take away some of their autonomy, I guess.” (MICU, 2)
	“I think for me, nudges, whether intentional or not intentional, it feels a little deceptive. Like, if you think one thing is right for a family and you feel strongly, you should just say that. You should say it out loud, and it should be more explicit, ‘These are the options, but I’d be lying if I didn’t say that I thought this option was the best one for these reasons.’ By nudging, it feels like if you are intentionally nudging, is it because what you think is best because of your own values or because of something more explicit, but you don’t want to say it outright? I find in general that families appreciate honesty…” (MICU, 51)
	“If I try to manipulate their decision, people are smart. They’re going to see it when they look back if it’s something that really didn’t fit their beliefs…I guess that’s why I try not to nudge, because I know that it’s not a long-term win at all.” (NICU, 50)
	“Yeah, I mean, I guess lightweight manipulation, which is otherwise known as persuasive argument or conversation, right, like where we draw the line of those things, I think it’s really hard sometimes, but yeah. I think it could be seen like that, depending on how forceful it is, the timing in which it’s done, and, you know, the intent behind it, I guess.” (MICU, 36)
	“And so the negative version when you steer too far one way is obviously coercion, and it’s on a spectrum, and I think we all agree almost in a tautology sense that coercion is bad. If you agree that something is coercion, coercion is bad, right? But I think where on that spectrum you are from nudging to coercion, if you’re on the nudging end, then you’re actually helping. You’re doing what patients come to physicians for, which is to help them feel good about making the best decision possible for themselves, and that’s why it is so dependent on the context and the situation and your relationship with a patient and their family and your prior conversations with them, et cetera, to know that you are helping guide them and not actually manipulating them in a pejorative sense of the word.” (PICU, 19)
Potential exacerbator of distrust	“I think certainly nudging, like all paternalistic practice, can certainly be unethical…I think nudging can be used certainly in a way that is unethical, in the way that a lot of biased care can be unethical.” (PICU, 21)
	“I think it’s fascinating is our own biases, if we’re unfamiliar with those religious texts or, you know, people’s different cultural values, whether nudging or other forms of covert mechanisms, it’s a way of kind of imparting your own biases whether you like it or not, and part of that, again, just comes with being human, but something that it’s good to be cognizant of.” (NICU, 52)
	“Sometimes nudges can make it more difficult because I think inherently there are a lot of patients from disadvantaged backgrounds or underrepresented backgrounds, oftentimes that have a distrust of the healthcare system, and I think it’s really important in those circumstances to be honest and try to understand where the family is coming from, because there is an inherent mistrust in what we recommend and maybe mistrust as to whether or not we’re providing the thing that’s best for the patient and best for the family.” (PICU, 12)
	“Yeah, I mean, in general, I try, especially in decisions like redirection of care, like I feel in most scenarios that is truly a family decision. If they want a recommendation based on that, you know, we can help support them, but in situations like that, I do think maintaining neutrality to the best of your ability and really nudges wouldn’t be appropriate, although I think a lot of people do. I personally do try to avoid bias and nudges.” (NICU, 46)
**Nudging may be better for some situations than others**	
How a nudge is delivered is important	“I don’t tend to bring in numbers around prognosis or hard numbers around prognosis, because I think sometimes they’re misleading…I think I would not want someone to try to make a decision with a misunderstanding of what the outcome is.” (MICU, 49)
	“I think that quoting made-up statistics is always inappropriate…I guarantee if we ever went back in the literature, we would not find a paper that remotely resembled the number we just stated, even if we really believed it because this person said it or that person said it all that time ago. Quoting numbers is not helpful.” (MICU, 17)
	“Certainly the sandwiching. When I consent patients for procedures, I talk through the risks and benefits, and I usually do this thing right at the beginning saying, ‘I wouldn’t recommend this procedure unless I felt that the benefits outweighed the risks.’” (MICU, 54)
	“Certainly if there is, you know, an epidemiologically common outcome in comparable situations…I would point out that most people in the situation elect to do X or Y or that our recommendation would be that in part, because that is what we do most of the time.” (MICU, 14)
It depends on what is at stake in the situation	“What’s challenging is that you don’t want to overstep when you’re the attending to say, ‘This is what we should do,’ because at the end of the day, it is a shared decision…it’s easier to provide a nudge rather than an overt recommendation, unless I feel like something grossly inappropriate is happening.” (MICU, 4)
	“I don’t try nudging, because they’re going to live with this for the rest of their lives…I just want them to be as well informed as possible, and depending on the context, I may not tell them what I think they should do.” (PICU, 47)
	“I think acuity, if there’s a family that won’t make a decision and it’s a time-sensitive decision…nudges have to be made, right? Because in some sense sitting there and not making a decision is making a decision…there’s kind of more ethical justification for using nudges.” (MICU, 34)
	“I think when the stakes are high, I probably maybe nudge less because I’m more explicit. You know, as you’ve described it, nudging is sort of a bit of a subliminal way of communicating such a message, but I actually don’t feel any shame or discomfort simply saying, ‘I think this is what we should do for the following reasons,’ and being a bit more prescriptive. Even in that, I think as the stakes increase, I am probably more prescriptive, and that’s not to say that anyone’s obligated to agree with my decision-making, but I probably move further towards more explicit forms of direction as opposed to nudging, if anything.” (MICU, 14)
Equipoise in decisions	“There’s this framework for informed consent…‘Here’s the recommendation. Here is the alternative,’ right? Except the truth is there isn’t really an alternative for some things….Actually, as the person responsible for administering this treatment, I’m not willing to take on that risk. That’s not actually your decision whether to take on that risk, so the truth is in some situations, there is no real alternative, and to pretend like there’s an alternative is doing a disservice…to pretend like there’s equipoise or to overwhelm them with statistics and stuff is not actually informed consent at that point.” (PICU, 19)
	“You know, I said it depends, like, I think acuity matters, yes, and the other thing I was going to say is that you don’t want to be manipulative about it, so if you really feel like there’s some way that is the right or wrong answer, then I think, you know, it’s fair to be upfront in there, but I think in areas where—again, this is where I say you have to recognize your own bias. A lot of times when we say there’s only 1 right answer, it’s because it’s our own bias necessarily that’s making it look like that, and I think where there’s real gray, that’s where it’s most effective.” (NICU, 53)
	“It’s a relatively narrow range of decisions where I feel like there’s so much medical certainty that I don’t necessarily even offer options. I sort of perform informed assents where I’m not really giving them a choice. I’m telling them what the plan is, and I’m giving them an opportunity to agree with me.” (MICU, 49)
Patient and family values	“I think it’s really important to understand the patient’s [family’s] values before you try to nudge them, so that if it is a really values-based decision, you can use these to nudge people toward what would best fulfill their goals and values…for the situations where I think we could actually only do harm and not benefit a patient, that wouldn’t be a situation where I would consider nudging.” (NICU, 52)
	“I think part of it goes to your relationship with the patient. I mean in transplant, we have a long-term relationship with many of our patients, so we really get to know them. In the ICU, you know, a typical medical ICU, you may only know the patient for a few hours or a couple of days. You may not have ever even spoken to them, just depending on their situation, and so you’re relying more on what the family’s saying. So I think, if you know the patient and have spoken with the patient and feel like you know their wishes, that nudging may be a little bit more appropriate, you know, if family seems to be veering away from that.” (MICU, 41)
	“I think more people who literally don’t know what to decide and they feel like they’re struggling even to grasp…to really get themselves on the pathway to making the decision, but there definitely also are people where I feel I have a sense of what they want, and they’re unable to state it in a way that actually is in line with something we can write down on paper as a medical decision…” (MICU, 26)
	“I mean I think it can be a good technique when you have a family who’s kind of, yeah, not understanding the situation or maybe coming at it where you maybe know enough about them to know they might choose differently, but they’re saying something different, and so it’s kind of filling a gap of like, how do I get you to understand what you need to.” (PICU, 4)

Abbreviations: ICU, intensive care unit; MICU, medical ICU; NICU, neonatal ICU; PICU, pediatric ICU.

**Table 3.** Trends Observed in Interviews Based on Physician Practice Setting and Sex

Concept	Practice setting			Sex	
	MICU	PICU	NICU	Female	Male
Use of nudging	Used primarily with families to guide decisions on aggressive interventions vs palliative care. Physicians framed options but emphasized best medical practice.	Strategically uses of nudging with parents, who are often key decision-makers. Physicians often guided parents toward safer or evidence-based choices without removing their agency.	Nudging was subtle and compassionate, often used to help families cope with difficult choices about premature infants or long-term care.	Often framed nudging as guidance rather than persuasion. Used empathetic communication to support autonomy. Emphasized patient/family emotional well-being and shared decision-making.	Were direct and authoritative in their nudging approach. Used data and medical authority to justify nudging. Frequently cited efficiency and clinical outcomes as the reason for nudging.
Ethical concerns	Concerned about decision fatigue in critically ill adults and whether nudging might push families toward physician-preferred options rather than patient wishes.	Ethical concerns arose around paternalism—how much to guide parents vs letting them make their own choices. Physicians worried about overstepping.	Focused on family autonomy due to long-term implications of NICU care. Nudging was seen as acceptable if it helped parents make informed decisions without feeling pressured.	Concerned about how nudging was perceived (eg, wanting to avoid being seen as pushy). Large emphasis on collaboration and emotional intelligence.	Focused on efficiency over how nudging might be perceived. Rarely saw autonomy as a major issue if nudging was based on clear medical evidence.
Examples	Conflicts often arose between physicians and families over the aggressiveness of treatment, particularly in end-of-life care.	Emotional distress of parents sometimes made nudging difficult. Physicians had to balance clinical guidance with family emotions.	Uncertainty of prognosis made nudging more delicate. Physicians were cautious about appearing too directive.	In cases of more clinical equipoise, emphasized patient and family emotions and concerns when guiding decisions.	In cases of more clinical equipoise, often cited medical statistics and previous clinical outcomes to steer parents toward a decision.

Abbreviations: MICU, medical intensive care unit; NICU, neonatal intensive care unit; PICU, pediatric intensive care unit.

### Nudging as a Part of Medical Communication

#### Physicians Responsibilities

Participants reported feeling a professional responsibility to nudge because they have the knowledge and ability to do so. They felt that patients expected their clinicians to steer them in medical decision-making, including via nudges (eg, one participant stated, “It’s part of the contract”), given their training backgrounds and experience with similar situations (eg, “Our job [is] to convey what we’ve seen,” as reported by another physician).

#### Role in Providing Care

Participants also felt that nudging was inherently part of their obligation to provide beneficent care. They felt that nudging helps patients and families navigate data and unknowns and act best on behalf of the patient. For example, one physician said, “[A physician’s] job is to help interpret data and help people navigate complex parts.” In doing so, they felt nudging can provide comfort to patients and families or normalize hard decisions, which is ultimately doing right by the patient and their families: “We give [families] permission to make the decision that seemed wrong…but is actually right.”

#### Trust With Patients and Families

Many participants felt that nudging was also integral to building a stronger physician-patient relationship and helped align medical recommendations with family values. One participant felt that nudges augment shared decision-making compared with strict recommendations because they take place in “gray areas when there is more than 1 reasonable approach.” Another participant said that by nudging, physicians are “guiding them and not actually manipulating them” and thus “helping them feel good about making the best decision possible for themselves.”

#### Existing Biases

Participants consistently felt that, because humans are always going to have biases, physicians should nudge to counteract those biases if they feel that these biases are irrational or inappropriate. For instance, when discussing end-of-life care for a patient who is terminally ill, one participant said, “Most people naturally will choose life…but what that means realistically down the road, I think many people don’t understand.” Others suggested nudging can also help patients become better informed without being overwhelmed by data.

#### Role in Decision-Making Process

Several participants argued that nudging could be a helpful tool for decision-making in acute situations with more clinical certainty or a clear clinical trajectory. One participant shared that when a patient is very ill, it is important to avoid laying out all possible options and instead “advocate as best for the patient” by nudging only reasonable and likely choices. Participants felt nudges that used defaults (such as presuming full code status), centered decisions around patient goals (eg, one physician reported, “I constantly reference what the patient would want”), and limited options (such as by offering a discrete set of options) helped make the decision-making process more efficient.

### Ethical Concerns About Nudges

#### Patient Autonomy and Informed Consent

Participants expressed concern that nudging may undermine patient autonomy, especially if it’s not transparent. One participant shared that if a nudge is too strong, “We risk making the patient’s decision for them.” Some shared nudging may infringe on informed consent (eg, a participant warned, “We risk leading them into a decision they don’t fully grasp”). Participants also highlighted that their concerns related to autonomy were especially prevalent in cases when there is less clinical certainty, such as those a participant described as “in gray areas, like palliative care decisions.” Some participants also noted that because physicians are only human and themselves biased, nudging in what they think is the best course of action can provide unhelpful direction. For instance, one participant stated, “Physicians tend to think quality of life is lower than [patients/families] perceive it”; thus, nudging in any direction could be wrong, since it may reflect the physicians’ values, not those of the patient or family. While the ethical questions raised by most physicians centered on concerns of potential deception or manipulation, at least 1 participant shared that a nudge could be potentially coercive if done too forcefully as a shove, where a patient or family feels undue pressure to pick a particular option.

#### Distrust With Patients and Families

Physicians consistently underscored the ethical concern that nudging could exacerbate distrust. Patients or families who are already skeptical of the medical system will “resist anything that feels like we’re pushing them in a direction,” said one physician. Another participant noted that “There’s potential for more abuse if we’re trying to impose our beliefs on them.” Because physicians “may be overly biased” by their own beliefs and cultural background, nudging could further hinder trust in subsequent encounters with the health care system: “If it’s something that really didn’t fit their beliefs…I try not to nudge because I know that it’s not a long-term win at all.” Some participants sensed that the deliberate use of nudges would bias care in a way that would worsen distrust with patients from already vulnerable populations.

### Appropriate Timing

#### Nudge Selection

Many participants expressed that the choice of nudges mattered. For instance, one participant differentiated between using a default option and “pointing out what most people in the situation elect” (ie, eliciting the bandwagon effect) to increase the uptake of a procedure because patients would be more aware of (and potentially more resistant to) the latter. Others felt that if the nudge was explicit and based on accurate information, then it would be more permissible. Using statistics to nudge, by contrast, was rarely appropriate according to participants because population-level statistics do not necessarily apply to individual patients.

#### The Stakes of the Situation

Participants noted that the appropriateness of a nudge also depends on the stakes of a situation. Specifically, in high-stakes scenarios, such as the decision to proceed with a tracheostomy, nudging may not be as appropriate: as one physician stated, “…they’re going to live with this for the rest of their lives.” Some participants believed that high-stakes situations necessitated more overt influence. For example, one participant expressed, “As the stakes increase, I am probably more prescriptive.” However, in more time-sensitive situations, such as during an acute decompensation, nudging might be helpful to prevent a delay. One participant noted, “Not making a decision is making a decision…there’s more ethical justification for using nudges.”

#### Equipoise in Decisions

When equipoise was present (ie, no clearly superior option or multiple reasonable options), many participants saw nudging as less appropriate. Where there is not a clear right choice, even in high-stakes situations (eg, redirection of care), “maintaining neutrality to the best of your ability” is most important and “nudges wouldn’t be appropriate,” as stated by one participant. This contrasts with situations with less equipoise and more evidence for a particular outcome. For example, one participant noted “In some situations, there is no real alternative, and to pretend like there’s an alternative is doing a disservice.”

#### Patient and Family Values

Participants noted that nudging is most appropriate when it could strengthen the physician-patient relationship or help align family values with medical recommendations. One participant described the situation as, “It’s kind of filling a gap…how do I get you to understand what you need to?” Nudging was deemed appropriate when physicians knew patients better: as a participant noted, “If you…feel like you know their wishes, nudging may be a bit more appropriate.” When patients or families had strong opinions or values, then nudging could further push families away.

### Physician Characteristics

#### Practice Settings

In general, intensivists in adult ICUs reported using more aggressive nudges that emphasized best medical practices, while pediatric and neonatal intensivists used more empathetic nudges (Table 3). One adult intensivist said, “[Families] often don’t understand how critical the situation is. Sometimes, nudging is necessary just to help them grasp reality.” A pediatric intensivist declared, “Parents are emotionally involved…you have to nudge gently, making them feel like they are part of the decision, even if you know what [is] best.” A neonatologist noted, “If I frame a decision too forcefully, [parents] might shut down. Instead, I say things like ‘many families in this situation find comfort in choosing palliative care,’ to guide them without pressure.”

#### Physician Sex

There were notable differences between female and male participants in the use of nudges (Table 3). In the interviews, female physicians often supported engaging in empathy-based nudging (eg, “I know this is difficult, but this option gives the best quality of life”), while male physicians frequently used data-driven nudging (eg, “This procedure has an 85% success rate”). A female participant noted she needs to “phrase my nudges as ‘something to think about,’ or I get more pushback from families.” Male physicians also frequently cited efficiency as reasons for nudging, while female physicians highlighted their use in facilitated shared decision-making; as one female physician put it, “I use nudging to make sure families don’t feel abandoned.”

#### Years of Experience

Younger physicians were typically more cautious about using nudges, citing ethical concerns regarding autonomy (Table 4). A junior physician acknowledged, “There’s a fine line between guiding and coercing.” In contrast, more senior physicians felt that nudging was a natural part of medical communication and were less concerned about ethical dilemmas, seeing nudges as inevitable and often beneficial: “After 20 years, I know which treatments are best. Nudging just makes sense.”

**Table 4.** Trends Observed in Interviews Based on Physician Experience

Concept	Experience, y		
	<10	10-19	>20
Views on nudging	More cautious about ethical concerns, emphasizing patient autonomy over persuasion. Avoided anything that could be seen as coercion.	Viewed nudging as a practical tool to help families make better decisions, especially when evidence was strong.	Saw nudging as a natural part of medical communication, often using subtle but effective ways to steer decisions.
Ethical concerns			
General	Concerned about whether nudging might be manipulative or overstep ethical boundaries.	Weighed ethical concerns but believed guidance was necessary for complex decisions.	Less concerned about ethical dilemmas; saw nudging as inevitable and often beneficial.
Related to autonomy	Concerned about overstepping. Preferred neutral framing to avoid manipulation or coercion.	Balanced nudging with shared decision-making. Acknowledged that some patients need guidance.	Less concerned about patient autonomy, saw nudging as a necessary part of good medical practice.
Related to informed consent	Emphasized fully informing the patient before any nudging occurred.	Used nudging alongside informed consent, ensuring patients felt involved.	Believed that informed consent was important but recognized that too much information could be overwhelming.
Related to emotional impact of nudging	Worried about how nudging might emotionally manipulate patients or families.	Used emotional framing selectively but remained cautious in high-stakes decisions.	Used emotional nudging as a deliberate strategy, particularly in palliative or end-of-life care.
Related to fairness in decision-making	Tried to present all options equally, even if one was clearly better.	Acknowledged true neutrality is impossible; framed options based on evidence and experience.	Accepted that framing recommendations is inevitable and believed that expertise should guide decisions.
Perceptions of ethical boundaries	Concerned about whether nudging compromises autonomy. Preferred neutral framing of options.	Viewed nudging as an essential tool but tried to balance influence with autonomy.	Believed nudging was ethical if the patient/family remained engaged in the decision.

## Discussion

Our qualitative study on the experiences and perspectives of nudges by critical care physicians identified multiple themes relating to the appropriate use and ethics of nudging patients in clinical decision-making. Many physicians described nudging as an inevitable and natural part of communication—but one that must be used thoughtfully for it to be ethically justifiable. Despite a growing philosophical literature focused on the ethics of nudging,^15,31,32,33,34,35,36,37,38,39^ there is limited research on how clinicians might consciously use nudges to influence their patients or grapple with ethical uncertainties.

Several studies propose ways to responsibly nudge in public health policy,^17,40,41,42^ but no consensus guidelines exist about whether, how or when physicians should engage in nudging patients in clinical decision-making. We used the themes and subthemes identified in our study to construct a framework for when nudges may be more appropriate (Box). We focused on the decision-making process and anticipated outcomes that philosophers and researchers in implementation science have incorporated into their existing models on the ethics of nudging.^17,20,43^ Importantly, there is likely no threshold for a single criterion that should determine whether use of a nudge is ethical, since much is dependent on context.^44,45^

Box. **Ethical Considerations for Nudges**

**Decision-Making Process**

A nudge is more justified when

- It is effective in counterbalancing existing biases to align patients with their interests.
- It is easy to pick alternative options and to resist.
- The physician values and considers the patient’s genuine interests.
- There is a trusting relationship between the physician and the patient.

**Anticipated Outcomes**

A nudge is more justified when

- The physician anticipates the outcome of a bad decision to be more harmful.
- It will predictably generate greater health benefits.
- The patient endorses the intended outcome.
- The medical decision or potential outcome has lower stakes.

Although nudges may be common in medicine,^22^ most participants in our study reported no formal training in nudging, including how or when it may be used. This finding is consistent with other studies showing that physicians lack training and competency in decision-making science, despite it being integral to their practice.^46,47^ Such a gap in training raises concern, since some nudges may be more effective than others (eg, defaults vs loss aversion framing).^48^ However, whether training physicians to use nudges leads to better patient outcomes requires further investigation.

Even within critical care, there is a lack of research addressing the use of nudges across adult, pediatric, and neonatal ICUs. For example, one study examined no escalation of treatment defaults in adult medical ICUs^49^ and another examined this in adult surgical ICUs,^50^ but to our knowledge, no studies have examined such defaults in pediatric or neonatal settings. Generally, research on the use of nudges in pediatric and neonatal critical care settings is more limited compared with those in adult medicine.^23,51^ Most studies on nudges in critical care have focused on physicians to try to foster different behaviors (eg, to promote serious illness conversations or improve enrollment in clinical trials),^52,53,54,55^ although a 2024 qualitative study examined physicians’ use of nudges in adult critical care.^56^ Our study describes how intensivists in adult, pediatric, and neonatal units all engage in nudging but may do so differently and with different ethical concerns.

Our study also described how perceptions of nudging may vary based on physician sex and years of experience. Female participants often reported framing nudges as guidance and endorsed empathetic, patient-centered communication, and they were generally more concerned with issues of autonomy compared with male participants. This is congruous with literature on gender differences in care and style that are thought to be related to different experiences and sexism in medicine.^57,58,59^ Additionally, our study demonstrated that younger physicians were more apprehensive about nudging due to concerns about autonomy and fairness. This is consistent with existing literature that documents how senior physicians may prefer more paternalistic approaches when engaging in shared decision-making with patients and families.^60,61,62^

There is little empiric research on physicians’ experiences and perspectives with nudging. Of the existing literature, one study found that while physicians viewed a nudge that pointed a critically ill patient with cancer toward hospice more favorably than a nudge toward chemotherapy, nonclinicians had the opposite perspective.^63^ Two other studies confirmed significant differences between physicians’ ethical attitudes toward clinical nudging and that of patients.^64,65^ This is an important finding, given research showing nudging can often result in failure to treat patients according to their own preferences^66,67,68,69^ and that patients prefer different amounts of autonomy.^70,71,72^ While our study did not involve nonclinical participants, continuing to better understand how both physicians and patients experience nudges is paramount to strengthening informed consent and physician-patient trust.

Our study has important implications for the practice of critical care. Given that there is little standardization in how physicians may approach sensitive conversations, present options, or discuss goals of care, critical care units could benefit from conducting internal reviews of how their clinicians engage in shared decision-making with patients and families. Focus groups conducted with relevant stakeholders, including patients, could help with such reviews. Although ICU physicians face particularly acute ethical challenges, some of the topics we investigated could also be explored in other clinical settings. Our hope is that the current study may serve as a guide for future research among practitioners from different specialties.

### Limitations

This study has several limitations. First, we recruited participants from 3 academic tertiary care centers in the same city. Transferability of the findings to other regions and other types of health systems, such as community hospitals and other nonacademic centers, may be thus limited. Second, participants practiced in intensive care, meaning their experiences and perceptions of nudging may differ from those in other specialties or settings (eg, outpatient). Third, this sample was predominantly White, and the findings may not be transferable to clinicians of different races and ethnicities. Fourth, we sought only clinician perspectives on nudging; themes were not informed by insights from patients or surrogate decision-makers. Fifth, some of the themes were at times contradictory; nudges can be helpful and problematic at the same time, and we hope our findings ignite further research on the nuances of nudges and their proper application.

## Conclusions

This qualitative study examines perspectives and concerns of intensive care physicians regarding the use of nudges in the decision-making process. Physicians influence patients in the decision-making process in many ways. Nudging patients is a well-known but poorly understood phenomenon. Our findings suggest that physicians may see nudging as an effective but ethically complex technique that requires careful thought when communicating with patients and families.

## Notes

Supplement 1. **eAppendix.** Semistructured Interview Guide

Supplement 2.
Data Sharing Statement
