# Supplementary material for: Critical Care Physicians’ Perspectives on Nudging in Communication
Source: JAMA Netw Open. 2025 Sep 10;8(9):e2531199. doi: 10.1001/jamanetworkopen.2025.31199 (PMC12423848; doi:10.1001/jamanetworkopen.2025.31199)
Supplement: Supplement 1. — eAppendix. Semistructured Interview Guide [file jamanetwopen-e2531199-s001.pdf]

Supplemental Online Content

Soled DR, Cummings CL, Berbert LM, et al. Critical care physicians' perspectives on nudging in communication. *JAMA Netw Open*. 2025;8(9):e2531199. doi:10.1001/jamanetworkopen.2025.31199

eAppendix. Semistructured Interview Guide

This supplemental material has been provided by the authors to give readers additional information about their work.

© 2025 Soled DR et al. *JAMA Network Open*.

eAppendix. Semistructured Interview Guide

Participant ID: __________________ Site:___________________
Date:_________________
Pre-Interview checklist

- □ Information Sheet emailed to participant
- □ Confirmed recording equipment present and working

Thank you again for meeting with me today. I really appreciate your meeting with us at this time, thank you for your willingness to participate. Your input is very valuable. This interview should take about 60 minutes. Feel free to stop me though at any time for any reason. I also wanted to confirm that you agree to having this study interview recorded, so that we make sure we have a complete and accurate record of our conversation. Is this okay with you?

1. First, we would like to ask you several demographics and characteristics questions. Can you please tell me the following: Do you work in a medical intensive care unit (or ICU), pediatric ICU, or neonatal ICU? What is your age? Excluding post-graduate training (i.e., residency and fellowship), how many years of experience do you have as an ICU attending? What is your sex? Please identify your race and ethnicity as one of the following: Asian, Black or African American, Hispanic, Middle Eastern, Multietnic, White, or Other (defined as an other race or ethnicity not previously mentioned).

2. The focus of this interview will be on patient decision-making in the ICU. Tell me about patient decision-making in the ICU. What kinds of challenges do you find? (Especially when patients are stuck between difficult options and are not sure what to do.)

   - How do patients make decisions? It is primarily decisions made by themselves or through surrogates?
   - What challenges do your patients face?
   - Is there ever really equipose?

3. How do you approach the presentation of options? Do you give thought into the timing and ways in which options are framed?

   - When are options presented to patients/surrogates? For instance, is it on rounds or during family meetings? Are options presented all at first then the risks of benefits of each, or are options presented followed by individuals risks and benefits?

4. What do you think about presenting choices with a default option that the patient / guardian would need to actively decline?

   - What are examples of defaults used in their ICU? Are defaults ever used with more “important” decisions?

© 2025 Soled DR et al. *JAMA Network Open*.

5. When you have a sense of a clearly better choice for the patient, how do you present those choices? Do you make specific recommendations? Do your biases possibly influence the way choices are presented? Describe an example for me if you can.

   - Does the timing or order of options change? Do you come up with your own idea of a "best" decision before presenting options to patients?
   - Do you ever try to obtain informed non-dissent, in which you as the physician bear the major burden of decision-making while ensuring that families retain the power to override any such decision?

6. Shared decision-making is when the patient and physician contribute to the medical decision-making process and agree on treatment decisions. What is your approach to achieving shared decision-making? How does this impact how you make recommendations?

   - What states is shared decision-making sought? For instance, is it equally sought with surrogates when a patient is intubated or incapacitated?
   - What situations is shared decision-making ever avoided?

7. Have you ever heard of the concept of "nudging"? The term has been defined as "any aspect of the choice architecture that alters people's behavior in a predictable way without forbidding any options or significantly changing their economic incentives. To count as a mere nudge, the intervention must be easy and cheap to avoid. Nudges are not mandates. Putting fruit at eye level counts as a nudge. Banning junk food does not." Nudging is distinct from making a specific recommendation; it is in between giving someone a simple menu of options and a mandate. In clinical conversations, nudging may manifest in how options are presented (for instance, the most "desirable" option first), whether any options are presented as the default (all patients are resuscitated unless their family chooses not to), choosing how to present risks and benefits of certain treatment options (for example, sandwiching the risks in between the benefits), citing figures to avoid losses (90% chance of survival versus 10% chance of death), or invoking what others in similar situations would do (for instance, most patients in this scenario would opt for X).

   - Have you ever had formal training on nudging? We define "formal" as attendance in at least one class or conference on decision-making science during or after medical school. Otherwise, have you ever had informal training on these topics? If so, what do you mean by informal?
   - Beauchamp and Childress present the following: Persuasion, manipulation, coercion. Persuasion is recommendations but you try to be neutral on the facts. Manipulation is when you're selectively presenting information (not necessarily lying) – giving information that will make patient more likely to pick one. Coercion is the use of a credible threat.
   - If necessary, can contrast three options: simple menu of options, nudges, and mandates.
   - Or put another way: Nudging is a continuum with a "recommendation" at one end of the continuum, followed by a mandate.
   - If yes, have you ever heard of it in healthcare? Has your department ever discussed it with you or other providers? Have you ever discussed it with house staff or trainees?

© 2025 Soled DR et al. *JAMA Network Open*.

- What are your thoughts about nudges in clinical conversations?
- When do you use nudges? Do you typically use them before presenting your recommendation or after?

8. Regardless of whether you make a specific recommendation, do you ever knowingly nudge patients to try to influence their decisions? In what ways?

   - What kinds of decisions might you have used nudging more? Are they typically more or less high-stakes decisions? Can you give an example?

9. Tell me about how you bring a patient's prognosis into your conversation when presenting medical options?

   - At what point does prognosis factor into your presentation of options and conversations around risks and benefits of particular options?

10. What do you think about using nudges to influence? Do you have any ethical concerns?

    - How does nudging relate to autonomy, justice, beneficience/non-maleficence?

11. Any thoughts on how nudges affect patient autonomy?

    - Do you think nudging impacts the patient's ability to voluntarily and freely decide particular options?

12. When might nudging be more appropriate or acceptable, compared to other more overt forms of influence such as making a specific recommendation?

    - Discuss in terms of the stakes and acuity of the decision.

13. In addition to nudges in clinical conversations, there may also be structural nudges in your unit to try to improve care. For instance, Foley catheters and A-lines being automatically removed after 3 days unless there are orders to the contrary, or antibiotics timing out after a certain amount of time. Does your unit have structural nudges?

    - Have you ever thought of these tactics as nudges?
    - What are your thoughts about structural nudges?

14. Do you have any other thoughts on decision-making, nudging, recommendations, or the like in your practice?

[Participants were then asked to respond to three hypothetical scenarios. These responses were not used for this paper's analysis and thus have been omitted from the supplement.]

© 2025 Soled DR et al. *JAMA Network Open*.
